# Sox10 Expressing Cells in the Lateral Wall of the Aged Mouse and Human Cochlea

**DOI:** 10.1371/journal.pone.0097389

**Published:** 2014-06-02

**Authors:** Xinping Hao, Yazhi Xing, Michael W. Moore, Jianning Zhang, Demin Han, Bradley A. Schulte, Judy R. Dubno, Hainan Lang

**Affiliations:** 1 Department of Otolaryngology – Head & Neck Surgery, Beijing Tongren Hospital, Capital Medical University, Beijing, China; 2 Department of Pathology and Laboratory Medicine, Medical University of South Carolina, Charleston, South Carolina, United States of America; 3 Department of Otolaryngology – Head & Neck Surgery, Medical University of South Carolina, Charleston, South Carolina, United States of America; 4 Department of Otolaryngology, Shanghai Yueyang Integrated Medicine Hospital, Shanghai, China; University of California, Irvine, United States of America

## Abstract

Age-related hearing loss (presbycusis) is a common human disorder, affecting one in three Americans aged 60 and over. Previous studies have shown that presbyacusis is associated with a loss of non-sensory cells in the cochlear lateral wall. Sox10 is a transcription factor crucial to the development and maintenance of neural crest-derived cells including some non-sensory cell types in the cochlea. Mutations of the Sox10 gene are known to cause various combinations of hearing loss and pigmentation defects in humans. This study investigated the potential relationship between Sox10 gene expression and pathological changes in the cochlear lateral wall of aged CBA/CaJ mice and human temporal bones from older donors. Cochlear tissues prepared from young adult (1–3 month-old) and aged (2–2.5 year-old) mice, and human temporal bone donors were examined using quantitative immunohistochemical analysis and transmission electron microscopy. Cells expressing Sox10 were present in the stria vascularis, outer sulcus and spiral prominence in mouse and human cochleas. The Sox10^+^ cell types included marginal and intermediate cells and outer sulcus cells, including those that border the scala media and those extending into root processes (root cells) in the spiral ligament. Quantitative analysis of immunostaining revealed a significant decrease in the number of Sox10^+^ marginal cells and outer sulcus cells in aged mice. Electron microscopic evaluation revealed degenerative alterations in the surviving Sox10^+^ cells in aged mice. Strial marginal cells in human cochleas from donors aged 87 and older showed only weak immunostaining for Sox10. Decreases in Sox10 expression levels and a loss of Sox10^+^ cells in both mouse and human aged ears suggests an important role of Sox10 in the maintenance of structural and functional integrity of the lateral wall. A loss of Sox10^+^ cells may also be associated with a decline in the repair capabilities of non-sensory cells in the aged ear.

## Introduction

The lateral wall of the cochlear duct is formed by the stria vascularis, outer sulcus, spiral prominence and spiral ligament. Structural and functional integrity of the cochlear lateral wall is required for generation of the highly positive endocochlear potential (EP) and maintenance of ion homeostasis in the inner ear [1]–[6]. A variety of lateral wall cell types play critical roles in the maintenance of the high K^+^ concentration and the positive EP in the scala media [5], [7], [8]. These cells and the ion transport mediators associated with their activity include 1) apical KCNQ1/KCNE1 channels and basolateral Na/K-ATPase and NKCC exchanger in strial marginal cells [9]–[11]; 2) Kir4.1 channel proteins in strial intermediate and outer sulcus root cells [12]–[14]; and 3) Na/K-ATPase, NKCC exchanger, Kir 5.1 channels, and carbonic anhydrase in fibrocytes of the spiral ligament [15]–[18].

Previous studies utilizing both animal models and human temporal bones have demonstrated that degeneration of cells in the cochlear lateral wall contributes significantly to age-related EP declines and auditory function deficits [1], [16], [19]–[27]. Unlike adult mammalian cochlear hair cells that lack the ability to regenerate, non-sensory cells in the cochlear lateral wall have been shown to have a limited regenerative capacity in response to injury [28]–[30]. However, the self-repairing capacity of cells in the cochlear lateral wall declines with age for unknown reasons [30], [31]. A better understanding of the cellular and molecular mechanisms associated with age-related cochlear lateral wall degeneration and declines in regenerative capacity is required to identify potential interventional strategies for the prevention and treatment of presbyacusis [32].

The neural crest (NC) is a transient developmental anlage arising at the edge of the neural plate in vertebrates. NC progenitor cells give rise to many cell types in the nervous system including strial intermediate cells in the cochlear lateral wall [33]. Sox10, a neural crest transcription factor that carries a conserved high-mobility group DNA-binding domain, is critical for the determination, differentiation and maintenance of peripheral glial cells and melanocytes [34]–[37]. Mutations of the Sox10 gene are known to cause degeneration and/or dysfunction of glial cells and melanocytes in a variety of tissues; e.g., Waardenburg syndrome in humans, a rare auditory-pigmentary disorder that generates varying combinations of hearing loss and pigmentation defects [38], [39]. Here, we investigated the potential role of Sox10 in the age-related degeneration of cells in the cochlear lateral wall by examining Sox10 immunostaining patterns in the inner ears of aged CBA/CaJ mice and human temporal bones from older donors.

## Materials and Methods

### Animals

Young adult and aged CBA/CaJ mice were used because this strain shows a larger decline in the EP with advanced age than other normal hearing mouse strains [40]. Adult CBA/CaJ mice were bred in-house in a low noise environment at the Animal Research Facility of the Medical University of South Carolina (MUSC). Original breeding pairs were purchased from The Jackson Laboratory (Bar Harbor, ME). All mice received food and water ad libitum and were maintained on a 12 h light/dark cycle. Mice of both genders including young adult mice aged 1–3 months (n = 12) and old mice aged 2–2.5 years (n = 15) were used in the study. All aspects of animal research were conducted in accordance with the guidelines of MUSC’s Institutional Animal Care and Use (IACUC) Committee. The protocol was approved by the IACUC at MUSC. The protocol number is #AR2290. Prior to data acquisition, mice were examined for signs of external ear canal obstruction and middle ear disease. Mice with any symptoms of ear infection were excluded from this study.

### Physiological Procedures

Mice (n = 27) were anesthetized by intraperitoneal injection with a mixture of xylazine (20 mg/kg) and ketamine (100 mg/kg) as described previously [41]. Auditory brainstem responses (ABRs) were recorded via customized needle electrodes inserted at the vertex (+) and test-side mastoid (−), with a ground in the control-side leg. The acoustic stimuli were generated using Tucker Davis Technologies system III modules (Tucker-Davis Technologies, Gainesville, FL, USA) with a SigGen software package. The calibration was completed using a Knowles microphone in a probe tube clipped to the mouse pinna. The signals were delivered into the mouse ear canal through a 10 mm long (3–5 mm diameter) plastic tube. ABR thresholds were obtained and were defined as the lowest sound levels at which the response peaks are clearly present as read by the eye from stacked wave forms. ABRs were evoked at half octave frequencies from 4 to 45 kHz with 5 ms duration tone pips with cos^2^ rise/fall times of 0.5 ms delivered at 31/s. Sound levels were reduced in 5-dB steps from 90 dB SPL to 10 dB SPL. At each sound level, 300–500 responses were averaged, using an “artifact reject” protocol whereby response waves were discarded when peak-to-peak amplitudes exceeded 50 mV. ABR waveforms and thresholds were analyzed at individual frequencies ranging from 4.0 to 40 kHz.

### Preparation of Mouse Cochlear Tissues

Following ABR measurements, young and aged CBA/CaJ mice were processed either for ultrastructural analysis (5 young and 6 aged) or immunohistochemical analysis (6 young and 8 aged). For ultrastructural observation, the anesthetized animals were perfused via cardiac catheter first with 10 ml of normal saline containing 0.1% sodium nitrite and then 15 ml of a mixture of 4% paraformaldehyde and 2% glutaraldehyde in 0.1 M phosphate buffer, pH 7.4. After removing the stapes and opening the oval and round windows, 0.5 ml of fixative was perfused gently into the scala vestibuli through the oval window. The inner ears were dissected free and immersed in fixative overnight (10–14 hours) at 4°C. Decalcification was completed by immersion in about 50 ml of a 120 mM solution of ethylenediaminetetraacetic acid (EDTA), pH 7.2, with gentle stirring at room temperature for 2–3 days with daily changes of the EDTA solution. For immunohistochemistry, the mouse inner ears were prepared following the procedure described above but substituting 4% paraformaldehyde as fixative for 1.5–2 hours at 4°C, decalcified with EDTA, cryoprotected in 30% sucrose in PBS and embedded in Tissue-Tek OCT compound (Electron Microscopy Science, Fort Washington, PA).

### Human Temporal Bone Collection and Cochlear Tissue Preparation

Temporal bones were selected from a collection of human temporal bones obtained as part of a longitudinal study of age-related hearing loss conducted by the Hearing Research Program at MUSC through partnership with the Carroll A. Campbell, Jr., Neuropathology Laboratory (Brain Bank) at MUSC. Procedures for the collection and use of the temporal bones were approved by the MUSC Institutional Review Board under protocol E-607R and Pro00030845, with written consent obtained in all cases. As shown in **Table 1**, the temporal bones used in this study were collected from 12 human donors. At autopsy, brain tissues including the brainstem and major blood vessels were carefully elevated from the cranial cavity. The temporal bones were removed using an oscillating 38-mm trephine saw according to previously described techniques [42]. Immediately following removal, the temporal bones were fixed by perilymphatic perfusion with 2 ml of a 4% paraformaldehyde solution (for frozen section preparations) or a 10% neutral buffered formalin solution (for paraffin section preparations). The pre-fixation process included elevation of the tympanic membrane, removal of the stapes and perforation of the round window membrane. The fixative was perfused gently through the oval window using a blunt-tip, 16-gauge needle covered with appropriately sized tygon tubing. The perfused temporal bones were then immersed in fixative for 48–72 hours for frozen section preparation and 12–22 hours for paraffin section preparation followed by rinsing and decalcification using a microwave protocol as per our previous description [43]. The total time of decalcification was between 3–6 weeks. During the process of decalcification, temporal bones were gradually trimmed using roungeurs and a No. 15 scalpel to remove most of the hard bone encasing the cochlea and vestibular apparatus.

**Table 1:** Summary of human donor information and immunoreactivity for Sox10 antibody.

ID number	DonorAge/Gender	Death to perfusioninterval	Sox10 immunostaining					
			OS	SP	SV (MC/IC)	AN	OCT	
**Frozen sections**								
H38R	68/female	6 hours 30 minutes	++	++	+/+	++		++
H44	72/female	Unknown	++	++	+/+	++		++
H31R	76/male	5 hours 45 minutes	++	++	+/+	++		++
H31L	76/male	5 hours 45 minutes	++	++	+/+	++		++
H55R	86/male	4 hours 15 minutes	++	++	+/+	++		++
H33R	87/female	3 hours 35 minutes	++	+	+/+	++		++
H36R	87/male	19 hours	+	+	−/+	++		+
H51R	89/female	11 hours	++	+	−/+	++		++
H39R	90/male	21 hours 30 minutes	+	+	−/−	++		++
H34R	91/female	3 hours 15 minutes	+	+	−/+	++		++
**Paraffin sections**								
H26	46/female	3 hours	-	-	−/−	–		–
H3	91/female	3 hours 15 minutes	-	-	−/−	–		–

Abbreviations: OS, outer sulcus; SP, spiral prominence; SV, stria vascularis; MC, marginal cell; IC, intermediate cell; AN, auditory nerve; OCT, organ of Corti.

### Immunohistochemical Analysis for Mouse and Human Cochlear Tissues

Human temporal bones were processed either for frozen or paraffin sectioning. Mouse cochlear tissues were prepared only for frozen sectioning. For frozen sectioning, cochlear tissues were cryoprotected in 30% sucrose in PBS and embedded in Tissue-Tek OCT compound (Electron Microscopy Science, Fort Washington, PA). The specimens were then sectioned serially in the horizontal plane at a thickness of 10 µm at −22 to −24°C, collected and mounted onto Colorfrost@ Plus microscope slides (Fisher Scientific, Pittsburgh, PA) for staining and storage at −20°C. For paraffin sectioning the decalcified inner ears were dehydrated and embedded in paraffin (Paraplast, XTRA, Oxford Labware, Sherwood Medical, St. Louis, MO). Serial sections were cut at a thickness of 4 µm and mounted two per slide on Colorfrost^®^Plus slides for staining and storage at room temperature. Every 25^th^ section was stained with hematoxylin and eosin (H & E) for the evaluation of morphological preservation.

Frozen or paraffin sections of cochlear tissue from mouse and human specimens were incubated overnight at 4°C with a primary antibody diluted in PBS. The primary antibodies used in this study were goat anti-Sox10 (1∶100, catalog no: sc17342, Santa Cruz, CA). The goat polyclonal antibody to Sox10 was raised against amino acids 1–50 of the N-terminal sequence of human Sox10 (manufacturer’s technical information). After two washes with 0.1 M PBS, the sections were incubated with an affinity purified biotinylated anti-goat IgG (1∶100; Vector Laboratories, Inc, CA catalog no: FI-5000) for 3 hours at room temperature, rinsed two times with 0.1 M PBS, and flooded with fluorescein avidin green (1∶80; Vector Laboratories, Inc, CA catalog: A-2011) for 1 hour at room temperature. Nuclei were then counterstained with propidium iodide (PI).

To further identify the cells expressing Sox10 in the cochlear lateral wall, dual immunostaining was performed for Sox10 and either Na, K-ATPase (1∶5000; 31b; gift from Dr. George Siegel, Department of Neurology, University of Michigan Medical School, Ann Arbor, MI; [44]) or carbonic anhydrase III (1∶200; [45]). The 31b antibody recognizes most known isoforms of Na, K-ATPase and reacts with marginal cells in the stria vascularis as well as type II and IV fibrocytes of the spiral ligament in mouse and human cochlear tissues [9], [17]. CAIII protein is an enzyme that in humans is encoded by the CA3 gene [46]. Immunoreactive CAIII has been localized in type I, III and IV fibrocytes in the gerbil inner ear [15], [45]. Quantitative analysis of Sox10^+^ cells was performed in the lateral wall of 6 ears from the young adult mouse group and 8 ears from the aged mouse group. Cell counts were conducted on 5 to 8 randomly selected 10 µm mid-modiolar frozen sections per mouse ear.

### Transmission Electron Microscopy

Cochlear tissues were post-fixed with 1% osmium tetroxide for 1 hour, dehydrated and embedded in Epon LX 112 resin. Semi-thin sections approximately 1 μm thick were cut and stained with toluidine blue. Ultra-thin sections were stained with uranyl acetate and lead citrate and examined by electron microscopy as previous described [41]. All three turns of the inner ears taken from 4 aged and 4 young control mice were examined in this study. Ten to 20 randomly selected sections from each mouse cochlea were examined. These sections included all three turns of the cochlear lateral wall.

### Confocal Microscopy and Image Presentation

The sections were examined either with a ZeissAxio Observer or a Zeiss LSM5 Pascal confocal microscope (Carl Zeiss, Inc.). The captured images were processed using Image Pro Plus software (Media Cybernetics, MD), AxioVison 4.8 (Carl Zeiss Inc.) and Zeiss LSM Image Browser version 2.0.70 (Carl ZeissInc.). Adobe Photoshop CS2 was employed to adjust brightness, contrast and sharpness of images with identical settings for all panels. Alterations were not performed on images used for quantitative purposes.

### Data Analysis

Unless otherwise specified, all data in the figures are presented as mean ± SEM. Data for Sox10^+^ cells were analyzed by two tailed, unpaired *t* test (SPSS, Chicago, IL). A value of *p*<0.05 was considered to be statistically significant.

## Results

**Figure 1:**
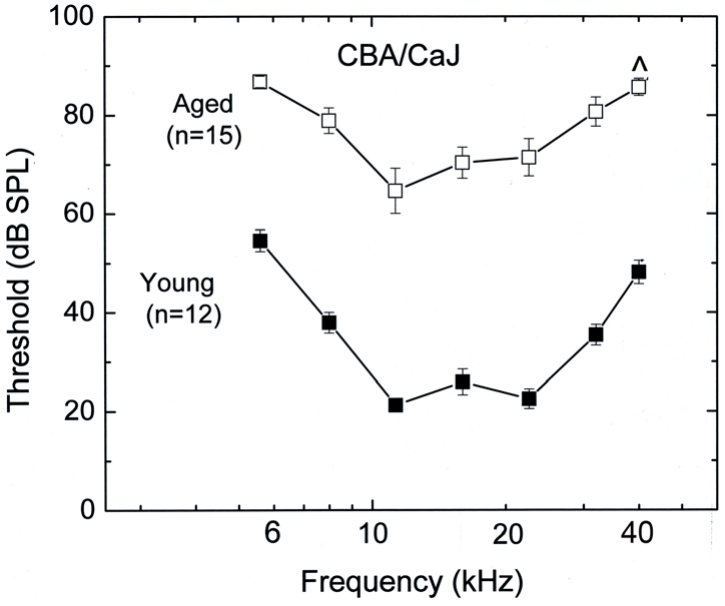
Auditory function declines with age in CBA/CaJ mice. ABR thresholds increased in aged mice at all tested frequencies. Mean ABR thresholds differed significantly between young adult (1–3 month-old) and aged (2–2.5 year-old) CBA/CaJ mice (mean ± SEM, *p*<0.05). Eight of the 15 aged mice (∧) showed no response to stimuli at 40 kHz.

Comparisons of the mean ABR thresholds for young adult (1–3 month-old; n = 12) and aged (2–2.5 year-old; n = 15) CBA/CaJ are shown in Figure 1. Relative to young adult controls, significant threshold shifts ranging from 20–35 dB were present in the aged mice at all frequencies tested (unpaired t-test, *P*<0.05).

**Figure 2:**
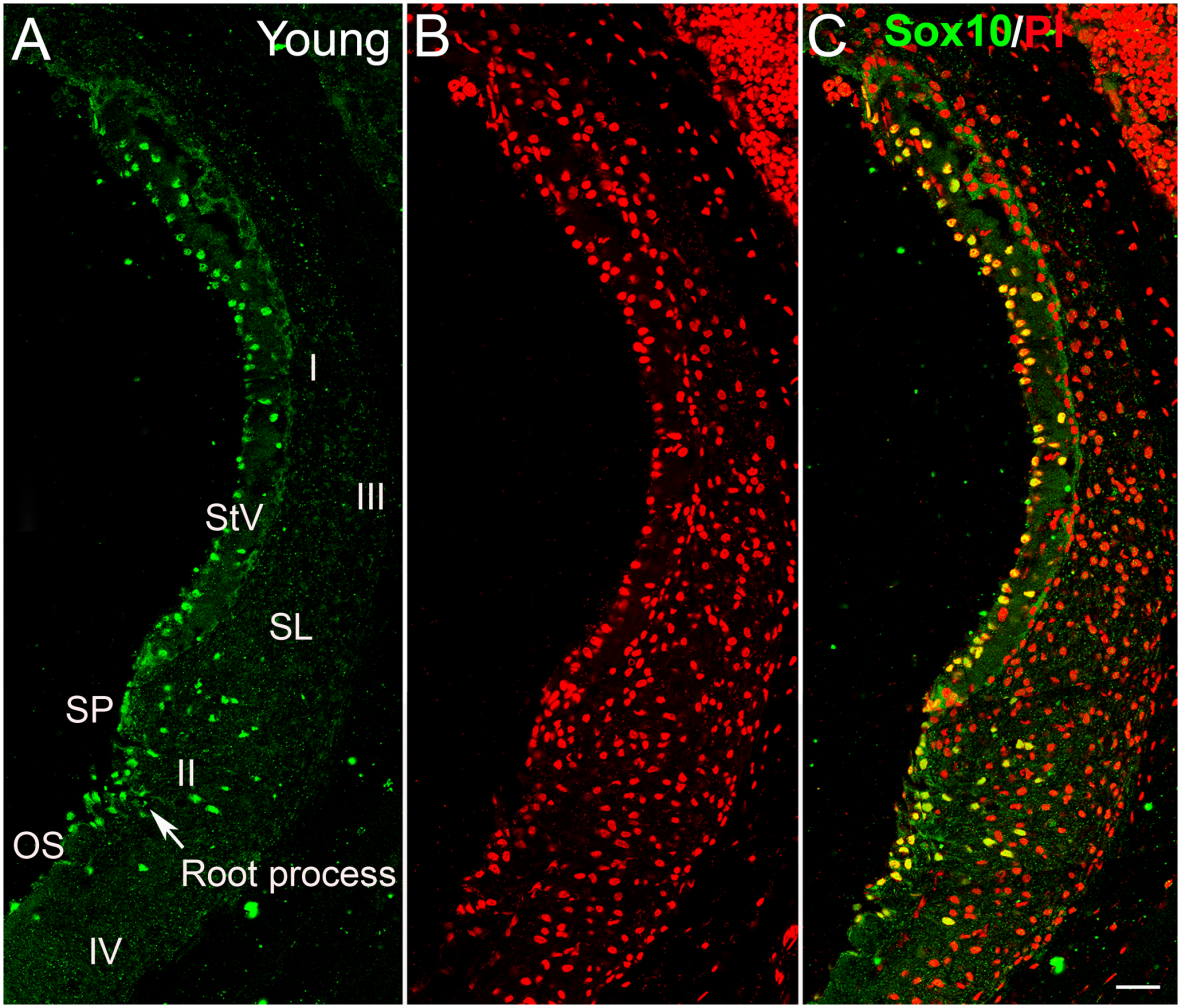
Localization of Sox10 expressing cells in the cochlear lateral wall of young adult mice. (A) Section from the middle turn of a 2 month-old mouse shows Sox10 expression (green) in the nuclei of marginal and intermediate cells in the stria vascularis (StV). The nuclei of both surface cells lining the scala media in the outer sulcus (OS) and root cells forming the root processes extending under the spiral prominence (SP) in the spiral ligament (SL) also were strongly positive for Sox10 staining. No Sox10^+^ cells were seen in regions occupied by type I, III and IV fibrocytes. (B) Nuclei were counterstained with propidium iodide (PI, red). (C) Merged image. Scale bar: 25 µm in C (applies to A–C).

**Figure 3:**
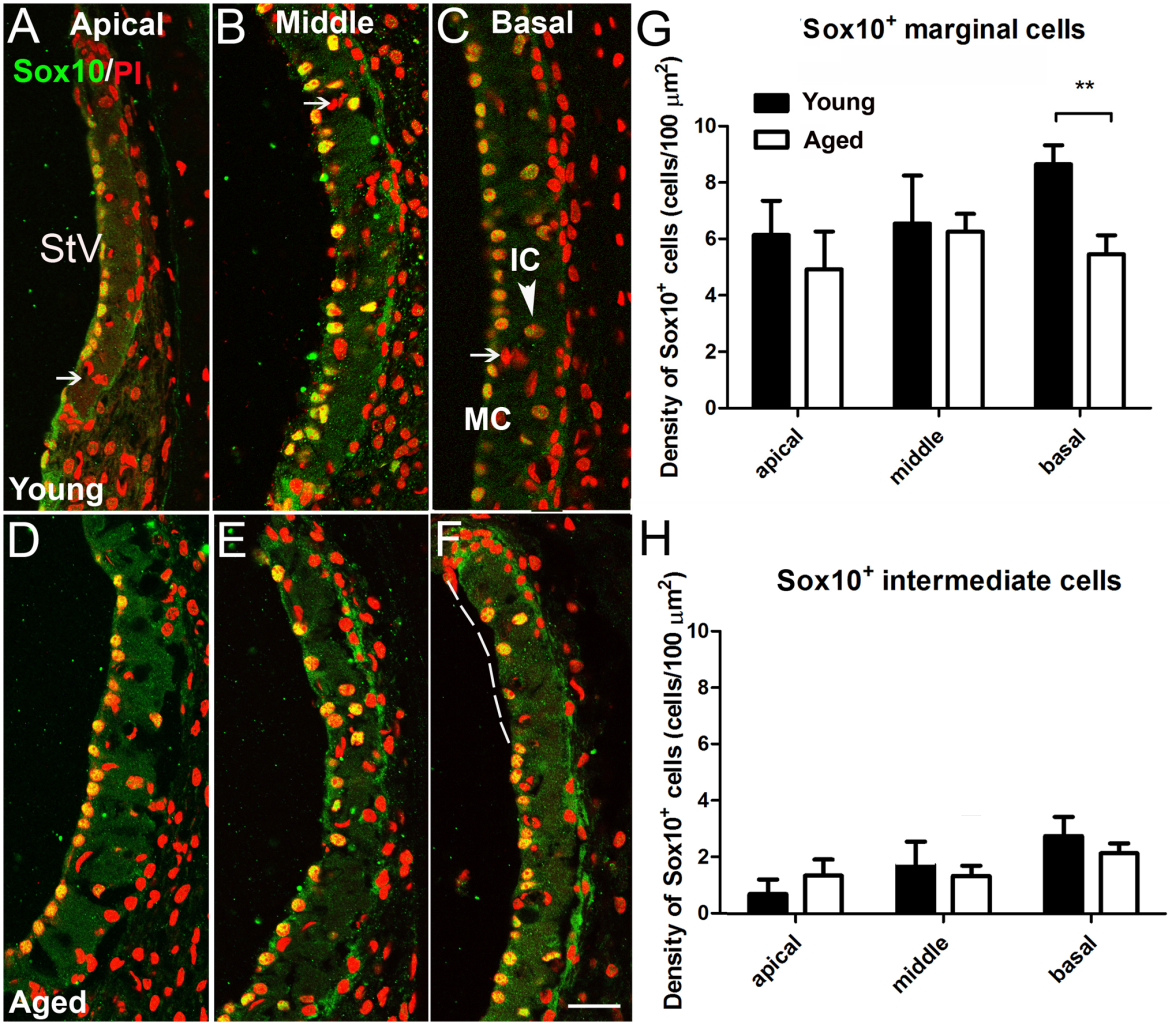
Age-related reduction of Sox10^+^ cells in the stria vascularis. Sox10^+^ marginal cells (MCs) and intermediate cells (ICs, arrowhead) were seen in the StV of young adult (A–C) and aged (D–F) mice. Endothelial cells and other cell types associated with strial microvascular structures were unstained with the Sox10 antibody (arrows). The dashed line in F indicates a region showing a complete loss of Sox10^+^ cells at the upper surface of the StV in the basal turn of an aged mouse. (G) Numbers of Sox10^+^ MCs decreased significantly in the basal turn of aged CBA/CaJ mice (n = 6, **p<0.01). Sox 10^+^ cells were also reduced in the apical turn, however, this difference was not significant. (H) Sox10^+^ ICs decreased in the middle and basal turns of aged ears, however, this reduction was not significant (n = 6, *p>0.05*). Scale bar: 20 µm in F (applies to A–F).

**Figure 4:**
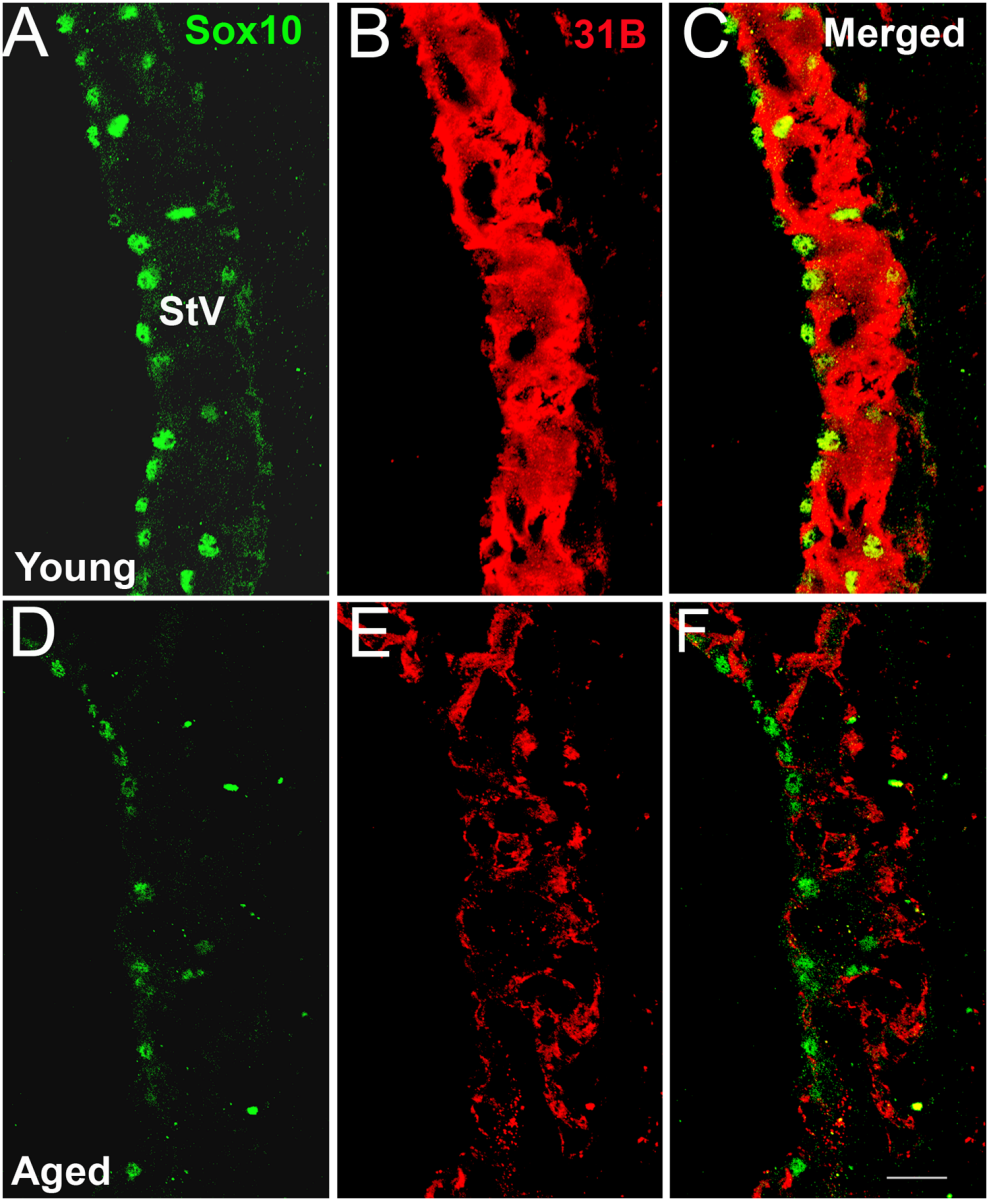
Age-related reduction of Na, K-ATPase immunoreactivity in the stria vascularis. Sox10 (green) and Na, K-ATPase (31B) immunoreactivity (red) in the StV of a young adult (A–C) and an aged (D–F) mouse. A dramatic decrease in 31B immunostaining intensity was accompanied by markedly decreased immunoreactivity for Sox10 seen in strial MCs in the aged mouse ear. Scale bar: 12 µm in B (applies to A–B’’).

**Figure 5:**
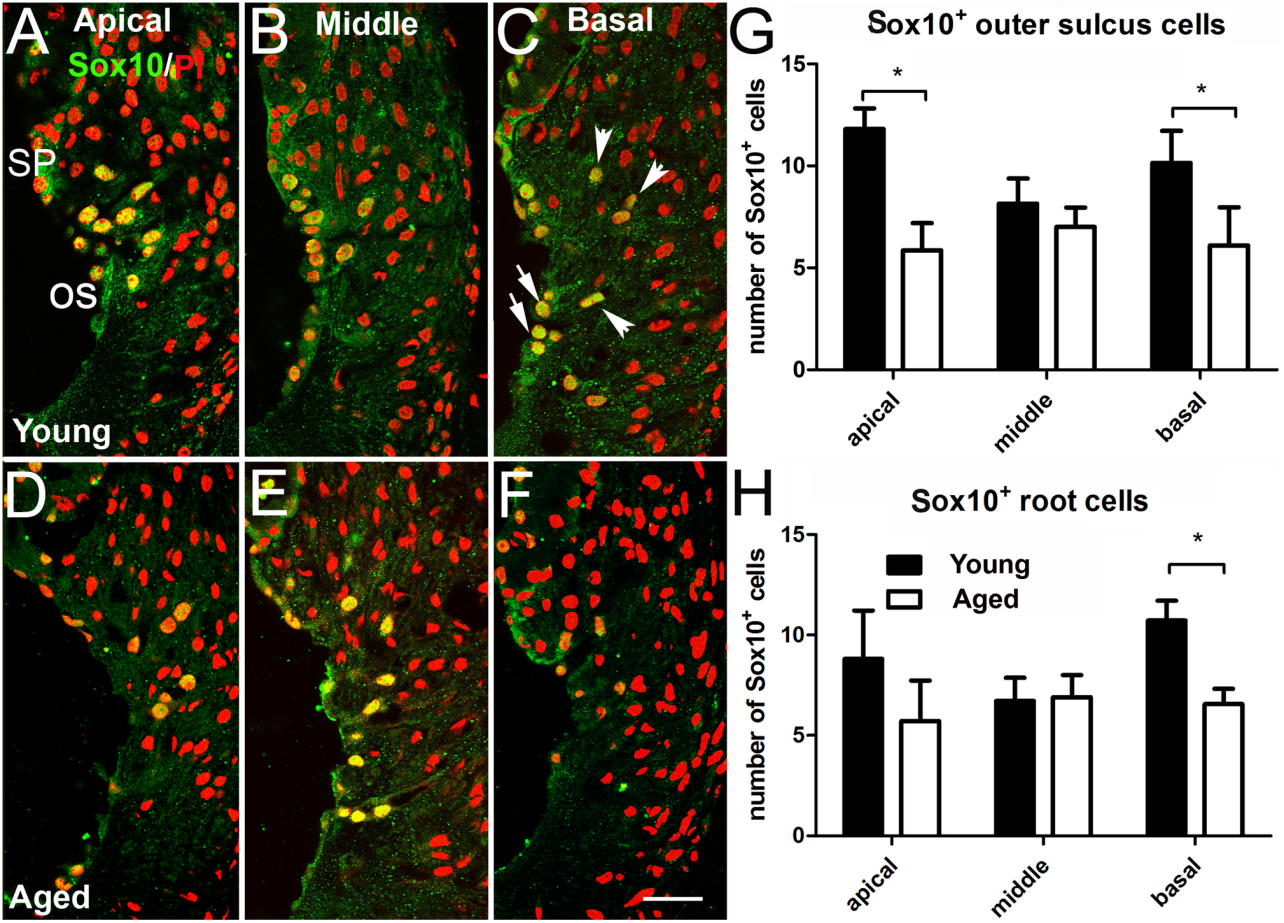
Age-related reduction of Sox10^+^ cells in the spiral ligament. Sox10^+^ superficial OS cells (arrows) and deep OS root cells (arrowheads) in young adult (A–C) and aged (D–F) mice. (G) Sox10^+^ superficial OS cells declined significantly with age in the apical and basal turns (n = 6, *p<0.05), but not in the middle turn. (H) Sox10^+^ root cells decreased significantly with age in the basal turn (n = 6, *p<0.05), but not in the apical and middle turns. Scale bar: 12 µm in F (applies to A–F).

A variety of cell types in the mammalian cochlear lateral wall have been characterized based on their location, morphology, immunostaining patterns for ion transporter mediators and cellular physiological characteristics [14], [15], [47]–[51]. These cells include marginal, intermediate and basal cells in the stria vascularis, outer sulcus cells including root cells extending into root processes in the spiral ligament, five types (I–V) of fibrocytes in the spiral ligament and various cell types constituting the microvasculature of the cochlear lateral wall. In this study, Sox10 expressing cells were present in several locations of the lateral wall including the outer sulcus, spiral prominence and stria vascularis (Figs. 2, 3, 4 and 5) in mouse ears. At least three cell types in the cochlear lateral wall were stained positively with antibody against Sox10: strial marginal and intermediate cells and outer sulcus cells, both those bordering the scale media (surface outer sulcus cells) and those extending deep into the outer sulcus root processes (root cells). Endothelial cells, pericytes and melanocytes (or macrophage-like melanocytes) form the strial microvascular system [51]. Few Sox10+ nuclei were located within or around the strial microvascular components, suggesting that few pericytes or macrophage-like melanocytes were stained positively with the antibody against Sox10. Several cell types in other cochlear locations in adult mouse ears also stained positively for Sox10. These cells included supporting cells in the organ of Corti and vestibular organs, glial cells in the auditory nerve and interdental cells in the limbus spiralis (data not shown).

### Age-related Changes of Sox10 Expressing Cells in the Stria Vascularis

Sox10 expressing cells were present in the stria vascularis of both young adult (Fig. 2) and aged CBA/CAJ mice (Fig. 3). Intense Sox10 immunoreactivity was present in the nuclei of marginal and intermediate cells, but not basal cells or any microvessel associated cells. To determine the effect of age on the populations of Sox10 expressing cells, cell counts were conducted on randomly selected areas covering a 100 µm-long segment of the stria vascularis and spiral prominence from all three cochlear turns in young adult and aged mouse ears. The average density of Sox10^+^ marginal cells in the stria vascularis of aged vs young mice was 5.7 vs 8.6/100 µm^2^ in the basal turn, 6.1 vs 6.5/100 µm^2^ in the middle turn and 4.9 vs 6.1/100 µm^2^ in the apical turn. This difference was significant (*p*<0.05) only in the basal turn (Fig. 3G). No significant decline in the density of Sox10^+^ intermediate cells was seen in any turn of the aged mouse ears (Fig. 3H).

Na, K-ATPase energizes the trans-epithelial movement of ions in many tissues. This enzyme is abundantly present in strial marginal cells and has been shown to decline markedly with age in quiet-aged gerbils [1], [9]. The relationship between Sox10 expression and strial ion transport functions was examined in sections from young adult and aged mice by dual-staining for Sox10 and Na, K-ATPase. As shown in Figure 4, a marked reduction of Na, K-ATPase immunoreactivity was seen in marginal cells in the basal turn of the aged mouse ear in regions of reduced or absent Sox 10^+^ immunoreactivity.

### Age-related Changes of Sox10 Expressing Cells in the Spiral Ligament

In the adult cochlea, outer sulcus cells line the surface of the scala media between Claudius cells and the spiral prominence. Some outer sulcus cells (root cells) elongate into root like processes that extend upward and behind the spiral prominence. These root processes are enclosed by a basement membrane and are most prominent in the basal turn and diminish in size and number toward the cochlear apex. The root processes are surrounded by and closely associated with type II fibrocytes. As shown in Figures 2 and 5, Sox10 immuoreactivity is present in the nuclei of both superficial outer sulcus cells and root cells, but not in the regions populated by type I–V fibrocytes (Figs. 2 and 5). Intense Sox10 immunostaining was seen in the nuclei of both superficial and deep root cells in the outer sulcus of both young adult and aged mice. All five types of fibrocytes were negative for Sox10 immunostaining.

Sox10^+^ superficial outer sulcus cells and root cells were counted in all three turns from young adult control (n = 6) and aged (n = 8) mouse ears (Fig. 5). Each section included the entire spiral prominence. The average number of superficial Sox10^+^ outer sulcus cells in aged and young ears respectively was 5.9 vs 11.8 per section in the apical turn, 7.1 vs 8.1 per section in the middle turn and 4.0 vs 10.1 per section in the basal turn. This difference was significant in the apical and basal turns but not in the middle turn (Fig. 5G; *p*<0.05). For root cells located deeper in the root process, the average Sox10^+^ root cell number per section in young adult and aged ears respectively was 5.7 vs 8.8 in the apical turn, 6.4 vs 6.7 in the middle turn, and 6.7 vs 10.7 in the basal turn. This decrease in Sox10^+^ root cells in aged mice was significant in the basal turn, but not in the apical and middle turns (Fig. 5H, *p*<0.05).

**Figure 6:**
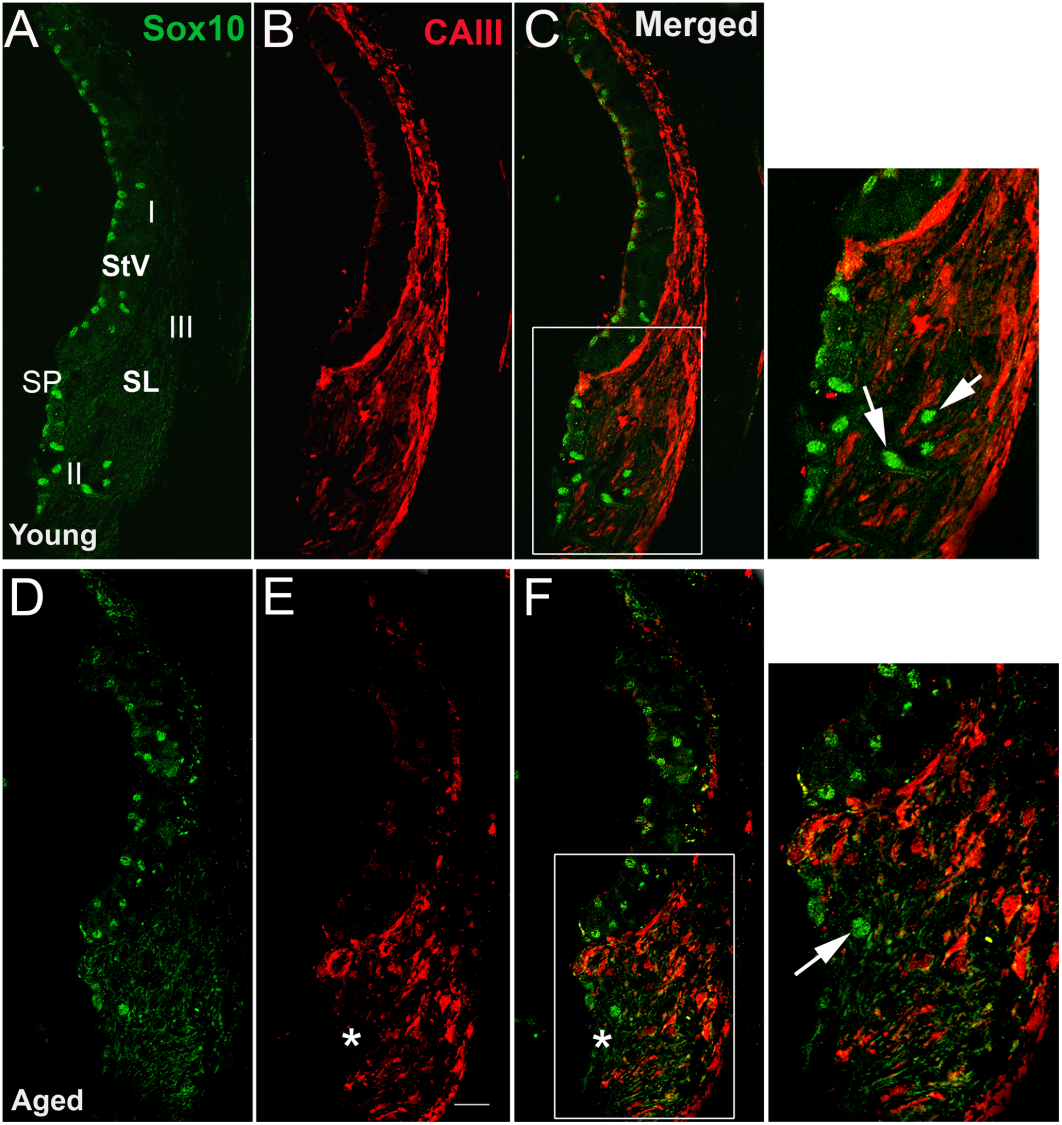
Age-related loss of carbonic anhydrase III immunoreactivity in the spiral ligament. Sox10 (green) and carbonic anhydrase (CA) III immunoreactivity (red) in the SL of a young adult (A–C) and an aged (D–F) mouse. CA III immunostaining intensity in areas occupied by types I and III fibrocytes declines markedly in aged ears. Far right panels show enlarged images of boxed areas in C and F. Nuclei of root cells (arrows) react strongly with Sox10 antibody in the young adult mouse but appear fragmented (arrow) or missing altogether in some root cells (asterisk) of aged mice. Scale bar = 12 µm in A (applies to A–B”).

Carbonic anhydrase (CA) has been detected in subpopulations of lateral wall fibrocytes in several species including human, chinchilla and gerbil suggesting the involvement of this enzyme in ionic or fluid regulation of the endolymph [15], [17], [52], [53]. To further confirm that no fibrocytes expressed detectable levels of Sox10, dual-immunostaining for Sox10^+^ and CAIII was performed on frozen sections of young adult and aged ears (Fig. 6). Similar to our previous findings young adult gerbils [15], intense immunostaining for CAIII was seen in type I and III fibrocytes in young adult mice. No Sox10 expressing cells were seen in these CAIII^+^ regions (Figs. 6 A–C). In aged mice, a remarkable reduction of CAIII immunoreactivity was present in type I and III fibrocytes (Figs. 6D–F’). Diffuse CAIII staining remained in the lower inferior of the type I fibrocyte areas where they border type II fibrocytes.

### Age-related Ultrastructural Changes in the Stria Vascularis and Root Process

**Figure 7:**
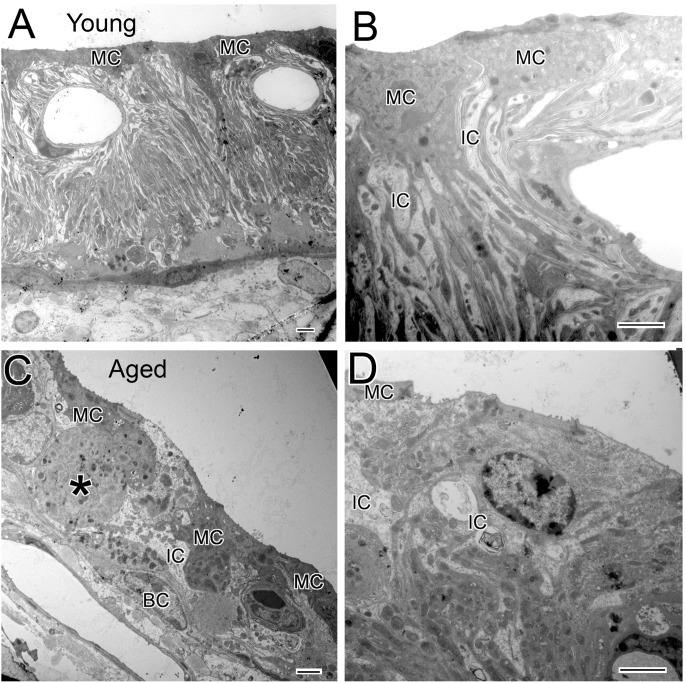
Age-related ultrastructural changes in the stria vascularis of CBA/CaJ mice. (A, B) Fine structure of strial marginal cells (MC) and intermediate-like cells (IC) from a young adult mouse. (C, D) Atrophic StV from an aged mouse. MCs and ICs appear to be in various stages of degeneration. A swollen region of a degenerating cell (asterisk) contains numerous melanophagosomes. Scale bars: 2 µm in A–D.

The ultrastructural characteristics of the stria vascularis in the young adult CBA/CaJ mouse are shown in Figure 7A and 7B. Multiple processes projecting from the basolateral plasmalemma of strial marginal cells interdigitate extensively with the more electron lucent processes of intermediate cells. Tight junctions between marginal cells form a barrier between endolymph and intrastrial fluid. Examination of aged mice revealed a range of degenerative alterations including: 1) loss or retraction of the long processes of marginal and intermediate cells; 2) appearance of melanophagosomes in degenerating marginal and intermediate cells; and 3) vacuoles and degenerative cellular debris in the cytoplasm of marginal and intermediate cells (Figs. 7C, D).

**Figure 8:**
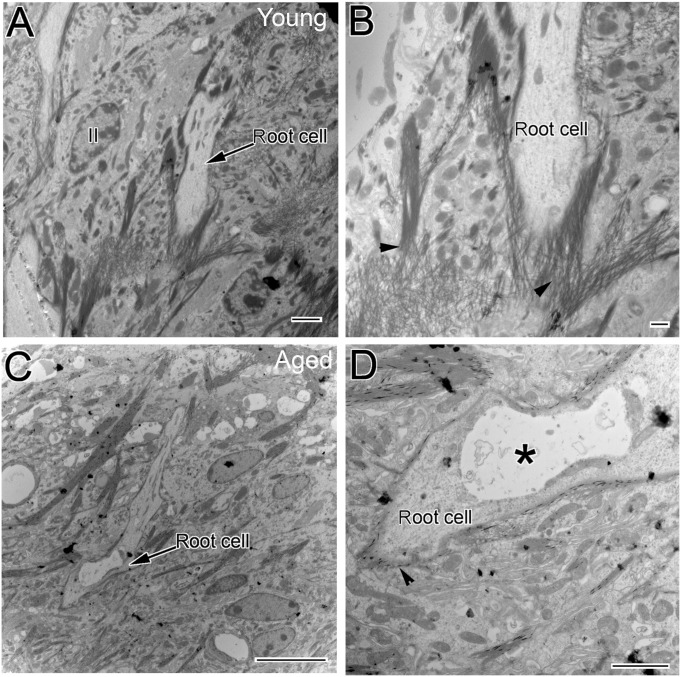
Age-related ultrastructural changes in the spiral ligament of CBA/CaJ mice. (A, B) Fine structure of the root cell processes (arrow) and surrounding type II fibrocytes (II) in the SL of a young adult mouse. Note that a thin fibril-enriched matrix is seen around the base of root cell processes. (C, D) Pathological alterations of root cell processes in an aged mouse. A substantial loss of the fibril-enriched matrix and a large edematous-appearing intracellular space (asterisk) is seen in a root cell. Scale bars: 2 µm in A; 500 nm in B; 10 µm in C, D.

Figures 8A and 8B depict the ultrastructure of root processes surrounded by type II fibcrocytes in the spiral ligament of a young mouse. A thin collagen fibril-enriched extracellular matrix is present around the basal portion of the root processes. Pathological alterations in aged cochleas include 1) a profound reduction of collagen-enriched matrix surrounding root processes and 2) the presence of vacuoles and degenerative cellular debris within root cells as well as the cytoplasm of type II fibrocytes (Figs. 8C, D).

### Sox10 Expressing Cells in the Human Cochlea

**Figure 9:**
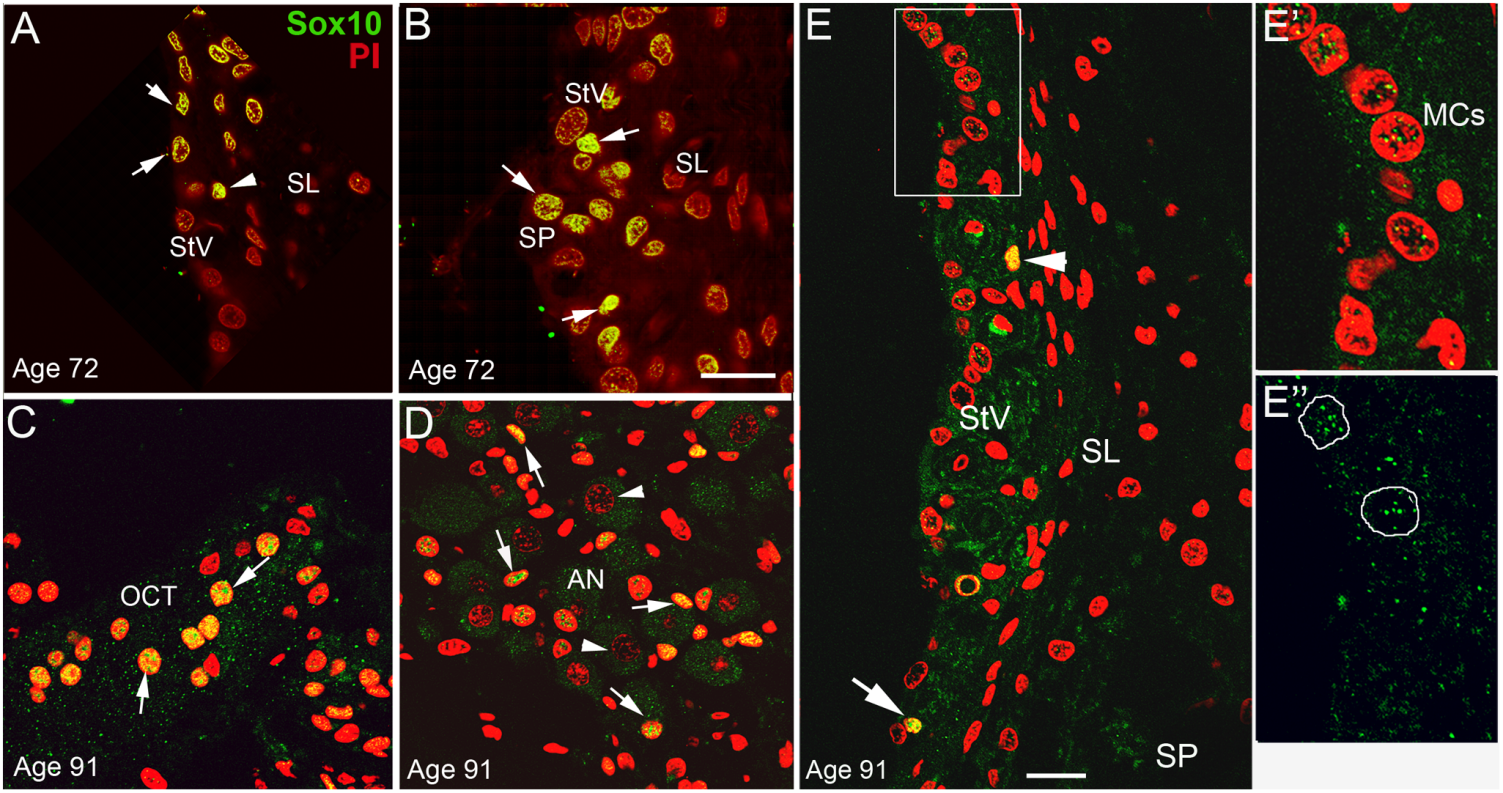
Sox10 expressing cells in human temporal bones. Cells staining positively for Sox10 were seen in several locations of the human cochlea including the lateral wall (A, B), the organ of Corti (OCT; C) and the auditory nerve (AN; D). The images shown are of apical and middle turn sections from a 72 year-old (A–B) and a 91 year-old (C–E). Moderate to strong immunostaining for Sox10 was seen in marginal cells (A, arrows), intermediate cells (arrowhead) and presumed root cells underlying the spiral prominence epithelium (B, arrows) in a 72 year-old donor, whereas only a few cells with weak immunoreactivity for Sox10 were seen in the StV (arrowhead; an intermediate cell) and in the SP region (arrow) in the cochlea of a 91 year-old donor (E). E’–E” are enlarged images of the boxed area in E showing the staining pattern of Sox10^+^ marginal cell nuclei. Scale bars: 12 µm in A (applies to A–B’’); 12 µm in E (applies to C–E).

A total of 12 temporal bones from 11 donors including 7 females and 5 males ranging from 46 to 91 year-old were utilized in this study (Table 1). Ten of the specimens were prepared for frozen sectioning and two were processed for paraffin sectioning. Seven temporal bones were harvested with a postmortem to fixation interval of less than 6 hours. Several locations in the inner ear portion of these human temporal bones were examined including the cochlear lateral wall, organ of Corti and auditory nerve. As shown in Figure 9, positive immunoreactivity for Sox10 in at least one cochlear location was seen in the frozen section preparations from all 10 temporal bones examined. Strong immunostaining for Sox10 was present in glial cells in the auditory nerve within Rosenthal’s canal, the osseous spiral lamina, and supporting cells in the organ of Corti (Figs. 9C, D). No immunostaining for Sox10 was seen in paraffin sections prepared from temporal bones despite the use of an antigen retrieval technique on more than 10 slides randomly selected from each ear.

Similar to our observations in the adult mouse cochlea, Sox10^+^ cells were seen in several regions of the human ear including the stria vascularis and outer sulcus including root cells (Figs. 9A–B, 9E–E’’). A marked reduction of Sox10 immunoreactivity was seen in marginal cells from the four oldest donors aged 89 to 91 as compared to donors aged 68 to 87. We were unable to perform quantitative analysis of age-related alterations of Sox10 expressing cells in the human cochlear lateral wall due to a paucity of young and middle-aged adult donors.

## Discussion

The stria vascularis and the outer sulcus epithelial cells contribute significantly to the generation and maintenance of the endocochlear potential (EP) and inner ear ion homeostasis [5], [9], [10], [14], [16], [54], [55]. The stria vascularis is composed of three cell types (marginal, intermediate and basal cells) and microvascular elements [47]–[50]. Endothelial cells, pericytes and melanocytes (or macrophage-like melanocytes) comprise the strial microvascular system [51], [56]. The outer sulcus, located between the spiral prominence and claudius cells, is lined by a pseudostratified epithelium consisting of a surface layer of cuboidal-shaped cells bordering the scala media and underlying root-like cells. The root cells interdigitate with one another and together form root processes which project laterally and superiorly into the spiral ligament underlying the spiral prominence. The root processes are enclosed by a basement membrane and are closely associated with capillary loops and surrounded by numerous type II fibrocytes. Recent electrophysiological evidence has demonstrated that root cells lining the outer sulcus are the essential component of the cochlear lateral wall and are critical for the generation and maintenance of cochlear ion gradients and the EP [14], [72].

Our data here show that Sox10 is expressed by several epithelial cell types in the cochlear lateral wall in both mouse and human specimens. We demonstrate for the first time strong staining with Sox10 antibody in the nuclei of strial marginal and intermediate cells as well as superficial and deep (root) cells in the outer sulcus in the adult mouse and human inner ear. These results suggest that the Sox 10 transcription factor may play an important role in regulating cochlear lateral wall function.

Sox10 transcription factor is a member of Sry-related high–mobility-group HMG box (Sox) family, which plays a key role in regulating the specification, determination, differentiation and maintenance of neural crest-derived cells [34], [35], [57]–[60]. Neural crest precursor cells have been shown to give rise to a wide range of cell types, including craniofacial chondrocytes, sensory and autonomic neurons, peripheral glial cells, and melanocytes including intermediate cells in the stria vascularis. Our findings have concurred with previous studies showing that the Sox10 gene is expressed in the stria vascularis, in particular in intermediate cells of postnatal mouse ears [61], [62]. The intense Sox10 immunoreactivity in strial intermediate cells of early postnatal and adult mice supports the neural crest derivation of intermediate cells as previously reported [63]. However, our results showing a high level of Sox10 expression in strial marginal and outer sulcus cells were surprising. These epithelial cells have not been reported to derive from neural crest, but rather are thought to be of epidermal lineage [64].

Transduction of sound to electrical signals by inner ear hair cells depends importantly on the regulation of extracellular potassium [k^+^] and other ions by a variety of cell types the in organ of Corti and lateral wall [3]. The supporting cells in the organ of Corti, which have previously been shown to express Sox10 [61], along with the outer sulcus cells in the cochlear lateral wall form a special gap junction-rich epithelial network, which contributes significantly to K^+^ recirculation back to endolymph [3], [14]. Sox 10 expression by all of the cells in this syncytial epithelial supporting cell network suggests that this transcription factor may be involved in the regulation of K^+^ recirculation and ion homeostasis in the cochlea. This supposition is supported by data showing that the Sox 10 transcription factor regulates expression of both connexin 32 and connexin 32 mRNA, a gap junction protein essential to the maintenance of ion homeostasis in the glial syncytium of the central nervous system [65].

A significant finding in this study was the reduced number of Sox10^+^ strial marginal and outer sulcus root cells with age in the mouse ear. The loss of Sox10-expressing non-sensory epithelial cells in the cochlear lateral with age is consistent with previous observations that degeneration of lateral wall non-sensory strial marginal and intermediate cells in the cochlear lateral wall contributes significantly to a reduction of the EP and associated age-related auditory functional declines [1], [16], [26], [66]. The age-related loss of Sox10^+^ strial marginal and outer sulcus root cells in mice was associated with pathological alterations in the surviving strial marginal and intermediate cells and fibrocytes of the spiral ligament similar to these seen in quiet-aged gerbils [20], [27]. The loss of retraction of primary and secondary marginal cell processes in aged mice almost certainly leads to a greatly reduced basolateral membrane surface area and resultant loss of membrane Na, K-ATPase, as shown here by a marked reduction in Na, K-ATPase immunoreactivity in the stria vascularis of aged mouse ears. Our data also showed that the age-associated loss of Sox10^+^ marginal and outer sulcus cells occurred predominately in the basal and/or apical turn, but not in the middle turn. These results suggest that Sox10^+^ cells in the middle turn may be more resistant to aging, a finding that agrees with previous studies in animal models of age-related hearing loss [1], [20], [40], [53], [67]–[70].

Ultrastructural evidence of fibrocyte degeneration (data not shown), similar to that seen in aging gerbils, was seen in the aged mouse spiral ligament as were changes in immunostaining pattern for CA III in spiral ligament fibrocytes of the aged mouse. Carbonic anhydrase affects ion movements in cells via rapid interconversion of water and carbon dioxide to bicarbonate and proton ions (or vice versa). Changes in CA II immunoreactivity have been reported in the aged gerbil lateral wall with several patterns varying from diminished to increased immunostaining [53]. As shown here, the moderate to strong immunostaining for CA III in type I and III fibrocytes in young adults was markedly decreased in the spiral ligament of aged mice. In addition, ultrastructural observations revealed alterations of the root process in aged mice including a loss of the fibril-enriched matrix at the base of the processes and the appearance of electron lucent inclusion bodies in the cytosol of the root cells. Together, the data show a loss of Sox10 expressing cells and ultrastructural alterations in the surviving Sox10^+^ cells in the spiral ligament of aged mice. These pathological alterations are likely to contribute to the disruption of K^+^ recycling and a decline in the EP, but the role of Sox 10 in this process remains unclear. Further molecular and functional studies of the interactions between the Sox10 transcription factor and key ion transport, ion channel and gap junction-associated factors are needed to better understand the initial causes of age-related non-sensory cell degeneration in the cochlear lateral wall.

Although the Sox10 expression changes in the mouse cochlear lateral wall reported here provide intriguing data concerning a role for Sox10 in the regulation of inner ear ion homeostasis, these experiments only provide a working model of changes in the aged human cochlea. To validate the expression pattern of Sox10 in a human model, we immunostained sections from 12 temporal bones; 10 prepared for frozen sectioning and 2 for paraffin sectioning. Strong immunoreactivity for Sox10, similar to that seen in the mouse model, was observed in several cell types in the stria vascularis, spiral prominence, organ of Corti and auditory nerve in the frozen sections. Moderately strong staining for Sox10 was present in strial marginal and intermediate cells and cells in the outer sulcus region from donors aged 68–87, but not in cochlear tissues from two donors aged 90 and 91, suggesting an age-related decline of Sox10 expression in these regions. No Sox10 immunoreactivity was observed in paraffin sections even after the application of antigen retrieval protocols indicating that paraffin embedding has a negative impact on the preservation of Sox10 immunoreactivity.

The study of human temporal bones is essential to furthering our knowledge of pathophysiological mechanisms in the human ear. Here, the Sox10 expression data demonstrated the utility of our temporal bone preparation procedure [43], [71] and immunostaining assay using a frozen sectioning process to identify a particular transcription factor with low expression in human postmortem cochlear tissues. Further quantitative studies of lateral wall degeneration associated with candidate regulatory factors (identified from animal models) in human temporal bones will significantly advance our understanding of the causes of presbyacusis and other auditory disorders.

## Conclusion

1. Sox10 protein is expressed in the nuclei of strial marginal and intermediate cells as well as outer sulcus epithelial cells in both young adult and aged CBA/CaJ mice.
2. Quantitative analysis of Sox10 expressing cells indicated a significant decline in Sox10^+^ cells in both the stria vascularis and outer sulcus in the basal turn of aged mice compared to young adult controls.
3. Ultrastructural observations in aged mice confirmed the degeneration of several cell types including strial marginal and intermediate cells, outer sulcus root cells and fibrocytes of the spiral ligament.
4. This study also provides immunohistochemical evidence of Sox10 expression in the human cochlea.
5. Decreases in Sox10 expression levels and a loss of Sox10^+^ cells with age in both mouse and human ears suggests an important role of Sox10 in the maintenance of structural and functional integrity of the cochlear lateral wall.

## References

[1] Schulte BA, Schmiedt RA (1992) Lateral wall Na, K-ATPase and endocochlear potentials decline with age in quiet-reared gerbils. Hear Res 61: 35–46. 1326507 10.1016/0378-5955(92)90034-k

[2] Schulte BA, Steel KP (1994) Expression of alpha and beta subunit isoforms of Na, K-ATPase in the mouse inner ear and changes with mutations at the Wv or Sld loci. Hear Res 78: 65–76. 7961179 10.1016/0378-5955(94)90045-0

[3] Spicer SS, Schulte BA (1998) Evidence for a medial K+ recycling pathway from inner hair cells. Hear Res 118: 1–12. 9606057 10.1016/s0378-5955(98)00006-9

[4] Minowa O, Ikeda K, Sugitani Y, Oshima T, Nakai S, et al (1999) Altered cochlear fibrocytes in a mouse model of DFN3 nonsyndromic deafness. Science 285: 1408–1411. 10464101 10.1126/science.285.5432.1408

[5] Hibino H, Kurachi Y (2006) Molecular and physiological bases of the K+ circulation in the mammalian inner ear. Physiology (Bethesda) 21: 336–345. 16990454 10.1152/physiol.00023.2006

[6] Wangemann P (2006) Supporting sensory transduction: cochlear fluid homeostasis and the endocochlear potential. J Physiol 576: 11–21. 16857713 10.1113/jphysiol.2006.112888 PMC1995626

[7] Mistrik P, Mullaley C, Mammano F, Ashmore J (2009) Three-dimensional current flow in a large-scale model of the cochlea and the mechanism of amplification of sound. J R Soc Interface 6: 279–291. 18682366 10.1098/rsif.2008.0201 PMC2659578

[8] Patuzzi R (2011) Ion flow in stria vascularis and the production and regulation of cochlear endolymph and the endolymphatic potential. Hear Res 277: 4–19. 21329750 10.1016/j.heares.2011.01.010

[9] Schulte BA, Adams JC (1989) Distribution of immunoreactive Na+, K+-ATPase in gerbil cochlea. J Histochem Cytochem 37: 127–134. 2536055 10.1177/37.2.2536055

[10] Wangemann P, Liu J, Marcus DC (1995) Ion transport mechanisms responsible for K+ secretion and the transepithelial voltage across marginal cells of stria vascularis in vitro. Hear Res 84: 19–29. 7642451 10.1016/0378-5955(95)00009-s

[11] Crouch JJ, Sakaguchi N, Lytle C, Schulte BA (1997) Immunohistochemical localization of the Na-K-Cl co-transporter (NKCC1) in the gerbil inner ear. J Histochem Cytochem 45: 773–778. 9199662 10.1177/002215549704500601

[12] Hibino H, Horio Y, Inanobe A, Doi K, Ito M, et al (1997) An ATP-dependent inwardly rectifying potassium channel, KAB-2 (Kir4. 1), in cochlear stria vascularis of inner ear: its specific subcellular localization and correlation with the formation of endocochlear potential. J Neurosci 17: 4711–4721. 9169531 10.1523/JNEUROSCI.17-12-04711.1997 PMC6573344

[13] Ando M, Takeuchi S (1999) Immunological identification of an inward rectifier K+ channel (Kir4.1) in the intermediate cell (melanocyte) of the cochlear stria vascularis of gerbils and rats. Cell Tissue Res 298: 179–183. 10555552 10.1007/s004419900066

[14] Jagger DJ, Nevill G, Forge A (2010) The Membrane Properties of Cochlear Root Cells are Consistent with Roles in Potassium Recirculation and Spatial Buffering. J Assoc Res Otolaryngol. 10.1007/s10162-010-0218-3 PMC2914247 20393778

[15] Spicer SS, Schulte BA (1991) Differentiation of inner ear fibrocytes according to their ion transport related activity. Hear Res 56: 53–64. 1663106 10.1016/0378-5955(91)90153-z

[16] Spicer SS, Schulte BA (1996) The fine structure of spiral ligament cells relates to ion return to the stria and varies with place-frequency. Hear Res 100: 80–100. 8922982 10.1016/0378-5955(96)00106-2

[17] Weber PC, Cunningham CD 3rd, Schulte BA (2001) Potassium recycling pathways in the human cochlea. Laryngoscope 111: 1156–1165. 11568535 10.1097/00005537-200107000-00006

[18] Hibino H, Higashi-Shingai K, Fujita A, Iwai K, Ishii M, et al (2004) Expression of an inwardly rectifying K+ channel, Kir5.1, in specific types of fibrocytes in the cochlear lateral wall suggests its functional importance in the establishment of endocochlear potential. Eur J Neurosci 19: 76–84. 14750965 10.1111/j.1460-9568.2004.03092.x

[19] Schuknecht HF, Gacek MR (1993) Cochlear pathology in presbycusis. Ann Otol Rhinol Laryngol 102: 1–16. 10.1177/00034894931020S101 8420477

[20] Spicer SS, Schulte BA (2002) Spiral ligament pathology in quiet-aged gerbils. Hear Res 172: 172–185. 12361880 10.1016/s0378-5955(02)00581-6

[21] Schmiedt RA, Mills JH, Boettcher FA (1996) Age-related loss of activity of auditory-nerve fibers. J Neurophysiol 76: 2799–2803. 8899648 10.1152/jn.1996.76.4.2799

[22] Wu T, Marcus DC (2003) Age-related changes in cochlear endolymphatic potassium and potential in CD-1 and CBA/CaJ mice. J Assoc Res Otolaryngol 4: 353–362. 14690053 10.1007/s10162-002-3026-6 PMC3202724

[23] Kusunoki T, Cureoglu S, Schachern PA, Baba K, Kariya S, et al (2004) Age-related histopathologic changes in the human cochlea: a temporal bone study. Otolaryngol Head Neck Surg 131: 897–903. 15577787 10.1016/j.otohns.2004.05.022

[24] Ohlemiller KK (2009) Mechanisms and genes in human strial presbycusis from animal models. Brain Res 1277: 70–83. 19285967 10.1016/j.brainres.2009.02.079 PMC2792931

[25] Ohlemiller KK, Lett JM, Gagnon PM (2006) Cellular correlates of age-related endocochlear potential reduction in a mouse model. Hear Res 220: 10–26. 16901664 10.1016/j.heares.2006.06.012

[26] Schmiedt RA (2010) The physiology of cochlear presbyacusis. In: The aging auditory system: perceptual characterization and neural bases of presbyacusis.; Gordon-Salant S, Frisina RD, Poper AN, Fry RR, editors. New York: Springer. xv, 301 p.

[27] Spicer SS, Schulte BA (2005) Pathologic changes of presbycusis begin in secondary processes and spread to primary processes of strial marginal cells. Hear Res 205: 225–240. 15953531 10.1016/j.heares.2005.03.022

[28] Ruben RJ (1967) Development of the inner ear of the mouse: a radioautographic study of terminal mitoses. Acta Otolaryngol: Suppl 220: 221–244. 6067797

[29] Forge A, Li L, Corwin JT, Nevill G (1993) Ultrastructural evidence for hair cell regeneration in the mammalian inner ear. Science 259: 1616–1619. 8456284 10.1126/science.8456284

[30] Lang H, Schulte BA, Schmiedt RA (2003) Effects of chronic furosemide treatment and age on cell division in the adult gerbil inner ear. J Assoc Res Otolaryngol 4: 164–175. 12943371 10.1007/s10162-002-2056-4 PMC3202712

[31] Lang H, Stevens SM, Xing Y, Havens LT, Hao X, et al (2013) Age-related alterations in neural crest-derived cells of the mouse inner ear. Assoc Res Otolaryngol Abstr 35: 905.

[32] Gates GA, Cooper JC Jr, Kannel WB, Miller NJ (1990) Hearing in the elderly: the Framingham cohort, 1983–1985. Part I. Basic audiometric test results. Ear Hear 11: 247–256. 2210098

[33] Hilding DA, Ginzberg RD (1977) Pigmentation of the stria vascularis. The contribution of neural crest melanocytes. Acta Otolaryngol 84: 24–37. 70955 10.3109/00016487709123939

[34] Herbarth B, Pingault V, Bondurand N, Kuhlbrodt K, Hermans-Borgmeyer I, et al (1998) Mutation of the Sry-related Sox10 gene in Dominant megacolon, a mouse model for human Hirschsprung disease. Proc Natl Acad Sci U S A 95: 5161–5165. 9560246 10.1073/pnas.95.9.5161 PMC20231

[35] Britsch S, Goerich DE, Riethmacher D, Peirano RI, Rossner M, et al (2001) The transcription factor Sox10 is a key regulator of peripheral glial development. Genes Dev 15: 66–78. 11156606 10.1101/gad.186601 PMC312607

[36] Kelsh RN, Raible DW (2002) Specification of zebrafish neural crest. Results Probl Cell Differ 40: 216–236. 12353478 10.1007/978-3-540-46041-1_11

[37] Finzsch M, Schreiner S, Kichko T, Reeh P, Tamm ER, et al (2010) Sox10 is required for Schwann cell identity and progression beyond the immature Schwann cell stage. J Cell Biol 189: 701–712. 20457761 10.1083/jcb.200912142 PMC2872908

[38] Read AP, Newton VE (1997) Waardenburg syndrome. J Med Genet 34: 656–665. 9279758 10.1136/jmg.34.8.656 PMC1051028

[39] Pingault V, Bondurand N, Kuhlbrodt K, Goerich DE, Prehu MO, et al (1998) SOX10 mutations in patients with Waardenburg-Hirschsprung disease. Nat Genet 18: 171–173. 9462749 10.1038/ng0298-171

[40] Ohlemiller KK, Dahl AR, Gagnon PM (2010) Divergent aging characteristics in CBA/J and CBA/CaJ mouse cochleae. J Assoc Res Otolaryngol 11: 605–623. 20706857 10.1007/s10162-010-0228-1 PMC2975886

[41] Lang H, Li M, Kilpatrick LA, Zhu J, Samuvel DJ, et al (2011) Sox2 up-regulation and glial cell proliferation following degeneration of spiral ganglion neurons in the adult mouse inner ear. J Assoc Res Otolaryngol 12: 151–171. 21061038 10.1007/s10162-010-0244-1 PMC3046328

[42] Schuknecht H (1968) Temporal bone removal at autopsy. Preparation and uses. Arch Otolaryngol 87: 129–137. 4865202 10.1001/archotol.1968.00760060131007

[43] Cunningham CD 3rd, Schulte BA, Bianchi LM, Weber PC, Schmiedt BN (2001) Microwave decalcification of human temporal bones. Laryngoscope 111: 278–282. 11210875 10.1097/00005537-200102000-00017

[44] Hieber V, Siegel GJ, Desmond T, Liu JL, Ernst SA (1989) Na, K-ATPase: comparison of the cellular localization of alpha-subunit mRNA and polypeptide in mouse cerebellum, retina, and kidney. J Neurosci Res 23: 9–20. 2545897 10.1002/jnr.490230103

[45] Spicer SS, Ge ZH, Tashian RE, Hazen-Martin DJ, Schulte BA (1990) Comparative distribution of carbonic anhydrase isozymes III and II in rodent tissues. Am J Anat 187: 55–64. 2105051 10.1002/aja.1001870107

[46] Lowe N, Edwards YH, Edwards M, Butterworth PH (1991) Physical mapping of the human carbonic anhydrase gene cluster on chromosome 8. Genomics 10: 882–888. 1916821 10.1016/0888-7543(91)90176-f

[47] Hinojosa R, Rodriguez-Echandia EL (1966) The fine structure of the stria vascularis of the cat inner ear. Am J Anat 118: 631–663. 5917201 10.1002/aja.1001180218

[48] Jahnke K (1975) [Intercellular junctions in the guinea pig stria vascularis as shown by freeze-etching (author's transl)]. Anat Embryol (Berl) 147: 189–201. 1180393 10.1007/BF00306733

[49] Anniko M, Nordemar H (1980) Embryogenesis of the inner ear. IV. Post-natal maturation of the secretory epithelia of the inner ear in correlation with the elemental composition in the endolymphatic space. Arch Otorhinolaryngol 229: 281–288. 6970572 10.1007/BF02565531

[50] Santos-Sacchi J (1982) An electronmicroscopic study of microtubules in the development of marginal cells of the mouse stria vascularis. Hear Res 6: 7–13. 7054137 10.1016/0378-5955(82)90003-x

[51] Neng L, Zhang F, Kachelmeier A, Shi X (2013) Endothelial cell, pericyte, and perivascular resident macrophage-type melanocyte interactions regulate cochlear intrastrial fluid-blood barrier permeability. J Assoc Res Otolaryngol 14: 175–85. 23247886 10.1007/s10162-012-0365-9 PMC3660918

[52] Lim DJ, Karabinas C, Trune DR (1983) Histochemical localization of carbonic anhydrase in the inner ear. Am J Otolaryngol 4: 33–42. 6424489 10.1016/s0196-0709(83)80005-2

[53] Spicer SS, Gratton MA, Schulte BA (1997) Expression patterns of ion transport enzymes in spiral ligament fibrocytes change in relation to strial atrophy in the aged gerbil cochlea. Hear Res 111: 93–102. 9307315 10.1016/s0378-5955(97)00097-x

[54] Salt AN, Melichar I, Thalmann R (1987) Mechanisms of endocochlear potential generation by stria vascularis. Laryngoscope 97: 984–991. 3613802

[55] Wangemann P (2002) K+ cycling and the endocochlear potential. Hear Res 165: 1–9. 12031509 10.1016/s0378-5955(02)00279-4

[56] Santi PA, Mancini P (2005) Cochlear Anatomy and Central Auditory Pathways. In: Otolaryngology head & neck surgery. Philadelphia, Pa.: Elsevier Mosby.

[57] Cheung M, Chaboissier MC, Mynett A, Hirst E, Schedl A, et al (2005) The transcriptional control of trunk neural crest induction, survival, and delamination. Dev Cell 8: 179–192. 15691760 10.1016/j.devcel.2004.12.010

[58] Southard-Smith EM, Kos L, Pavan WJ (1998) Sox10 mutation disrupts neural crest development in Dom Hirschsprung mouse model. Nat Genet 18: 60–64. 9425902 10.1038/ng0198-60

[59] Haldin CE, LaBonne C (2010) SoxE factors as multifunctional neural crest regulatory factors. Int J Biochem Cell Biol 42: 441–444. 19931641 10.1016/j.biocel.2009.11.014 PMC2826508

[60] Stolt CC, Wegner M (2010) SoxE function in vertebrate nervous system development. Int J Biochem Cell Biol 42: 437–440. 19647093 10.1016/j.biocel.2009.07.014

[61] Watanabe K, Takeda K, Katori Y, Ikeda K, Oshima T, et al (2000) Expression of the Sox10 gene during mouse inner ear development. Brain Res Mol Brain Res 84: 141–145. 11113541 10.1016/s0169-328x(00)00236-9

[62] Wakaoka T, Motohashi T, Hayashi H, Kuze B, Aoki M, et al (2013) Tracing Sox10-expressing cells elucidates the dynamic development of the mouse inner ear. Hear Res 302: 17–25. 23684581 10.1016/j.heares.2013.05.003

[63] Steel KP (1999) Perspectives: biomedicine. The benefits of recycling. Science 285: 1363–1364. 10490411 10.1126/science.285.5432.1363

[64] Kim HM, Wangemann P (2011) Epithelial cell stretching and luminal acidification lead to a retarded development of stria vascularis and deafness in mice lacking pendrin. PLoS One 6: e17949. 21423764 10.1371/journal.pone.0017949 PMC3056798

[65] Schlierf B, Werner T, Glaser G, Wegner M (2006) Expression of connexin47 in oligodendrocytes is regulated by the Sox10 transcription factor. J Mol Biol 361: 11–21. 16822525 10.1016/j.jmb.2006.05.072

[66] Lang H, Jyothi V, Smythe NM, Dubno JR, Schulte BA, et al (2010) Chronic reduction of endocochlear potential reduces auditory nerve activity: further confirmation of an animal model of metabolic presbyacusis. J Assoc Res Otolaryngol 11: 419–434. 20372958 10.1007/s10162-010-0214-7 PMC2914241

[67] Thomopoulos GN, Spicer SS, Gratton MA, Schulte BA (1997) Age-related thickening of basement membrane in stria vascularis capillaries. Hear Res 111: 31–41. 9307309 10.1016/s0378-5955(97)00080-4

[68] Lang H, Schulte BA, Zhou D, Smythe N, Spicer SS, et al (2006) Nuclear factor kappaB deficiency is associated with auditory nerve degeneration and increased noise-induced hearing loss. J Neurosci 26: 3541–3550. 16571762 10.1523/JNEUROSCI.2488-05.2006 PMC2897814

[69] Sha SH, Kanicki A, Dootz G, Talaska AE, Halsey K, et al (2008) Age-related auditory pathology in the CBA/J mouse. Hear Res 243: 87–94. 18573325 10.1016/j.heares.2008.06.001 PMC2577824

[70] Hequembourg S, Liberman MC (2001) Spiral ligament pathology: a major aspect of age-related cochlear degeneration in C57BL/6 mice. J Assoc Res Otolaryngol 2: 118–129. 11550522 10.1007/s101620010075 PMC3201181

[71] Xing Y, Samuvel DJ, Stevens SM, Dubno JR, Schulte BA, et al (2012) Age-related changes of myelin basic protein in mouse and human auditory nerve. PLoS One 7: e34500. 22496821 10.1371/journal.pone.0034500 PMC3320625

[72] Jagger DJ, Forge A (2012) The enigmatic root cell - emerging roles contributing to fluid homeostasis within the cochlear outer sulcus. Hear Res 303: 1–11. 23151402 10.1016/j.heares.2012.10.010

